# Multiple Leiomyomata

**DOI:** 10.1155/S1064744997000550

**Published:** 1997

**Authors:** Sebastian Faro

**Affiliations:** Department of Obstetrics and Gynecology Rush-Presbyterian-St. Luke's Medical Center 1653 West Congress Parkway Chicago IL USA


					Infectious Diseases in Obstetrics and Gynecology 5:318 (1997)
(C) 1998 Wiley-Liss, Inc.
Images in Infectious Diseases in Obstetrics and Gynecology
Section Editor: David E. Soper, MD
Multiple Leiomyomata
Sebastian Faro, MD, PhD
Department of Obstetrics and Gynecology,
Rush-Presbyterian-St. Luke's Medical Center, Chicago, IL
LEGEND TO IMAGE
(A) Uterus with multiple leiomyomata. Patient pre-
sented with acute abdomen. Upon entering the abdo-
men the patient was found to have one to two liters of
purulent fluid. (B) Necrotic leiomyomata that had para-
sitized the transverse colon and eroded into the lumen of
the bowel. The patient required a total abdominal hys-
terectomy with bilateral salpingo-oophorectomy, bowel
resection, and colostomy. (C) 1998 Wiley-Liss, Inc.
B
Images in Infectious Diseases in Obstetrics and Gynecolo presents clinically important visual images that a practitioner in women's health
might encounter. If you have a high-quality color or black-and-white photograph or slide representing such an image that you would
like considered for publication, send it with a descriptive legend to David E. Soper, MD, Department of Obstetrics and Gynecology,
Rush-Presbyterian-St. Luke's Medical Center, 1653 West Congress Parkway, Chicago, IL 60612-3833.
Images is made possible through an educational grant from 3M Pharmaceuticals.
Received 2l January 1998
Accepted 6 February 1998

				

